# Human adipose-derived mesenchymal stem cells seeded into a collagen-hydroxyapatite scaffold promote bone augmentation after implantation in the mouse

**DOI:** 10.1038/s41598-017-07672-0

**Published:** 2017-08-02

**Authors:** Giovanna Calabrese, Raffaella Giuffrida, Stefano Forte, Claudia Fabbi, Elisa Figallo, Lucia Salvatorelli, Lorenzo Memeo, Rosalba Parenti, Massimo Gulisano, Rosario Gulino

**Affiliations:** 1 IOM Ricerca, Viagrande, (CT) Italy; 2 Fin-Ceramica Faenza, Faenza, (RA) Italy; 3 Department of Biomedical and Biotechnological Sciences, University of Catania, Catania, Italy; 4 Department of Medical and Surgical Sciences and Advanced Technologies, G.F. Ingrassia, “Policlinico Vittorio Emanuele”, Anatomic Pathology Section, University of Catania, Catania, Italy; 5 Department of Experimental Oncology, Mediterranean Institute of Oncology, Viagrande, Italy

## Abstract

Traumatic injury or surgical excision of diseased bone tissue usually require the reconstruction of large bone defects unable to heal spontaneously, especially in older individuals. This is a big challenge requiring the development of biomaterials mimicking the bone structure and capable of inducing the right commitment of cells seeded within the scaffold. In particular, given their properties and large availability, the human adipose-derived stem cells are considered as the better candidate for autologous cell transplantation. In order to evaluate the regenerative potential of these cells along with an osteoinductive biomaterial, we have used collagen/hydroxyapatite scaffolds to test ectopic bone formation after subcutaneous implantation in mice. The process was analysed both *in vivo*, by Fluorescent Molecular Tomography (FMT), and *ex vivo*, to evaluate the formation of bone and vascular structures. The results have shown that the biomaterial could itself be able of promoting differentiation of host cells and bone formation, probably by means of its intrinsic chemical and structural properties, namely the microenvironment. However, when charged with human mesenchymal stem cells, the ectopic bone formation within the scaffold was increased. We believe that these results represent an important advancement in the field of bone physiology, as well as in regenerative medicine.

## Introduction

Traumatic injury or surgical excision of infected or neoplastic bone tissue would require the reconstruction of large bone defects that will not heal spontaneously, especially in older individuals. The successful bone repair frequently requires tissue graft strategies to restore the anatomical and functional status of the tissue. Different transplantation approaches have been tried so far. For instance, de-vitalized allografts from cadaveric sources have been used, but they have shown a low osteogenic potential and a major risk of infection^1, 2^. Autologous bone transplantation still represents the gold standard due to higher osteogenic potential of bone implants and the absence of immune reaction. However, a moderate risk of pain and infection at the site of bone harvesting still exists^3, 4^. Therefore, bone tissue engineering represents a promising alternative and its importance has increased during the last decade^5^. The field of bone tissue engineering relies on the development of novel biomaterials, capable of mimicking native bone structure in terms of both mechanical and osteoinductive properties^6–12^. Moreover, these materials should also have angiogenic capability to improve the clinical success rate of bone repair^13–15^. The successful ossification of the scaffold is linked to its physical and chemical properties, and to the ability to incorporate cells and induce their correct differentiation^5, 13, 16–18^. The cells invading an implanted scaffold could come from the host tissues or they could be seeded on it before implantation^1, 7, 9, 10, 12, 16, 19–23^. Among the available exogenous cells, the human adipose-derived stem cells (hADSCs) have demonstrated higher capacity of proliferation and multi-lineage differentiation *in vitro* ^24, 25^, and they are considered the most attractive mesenchymal stem cells (MSCs). These properties, along with their ease of withdrawal and large availability make these cells as the better candidate for autologous cell transplantation^6, 14, 24–29^.

Wide varieties of synthetic or natural biomaterials have been employed so far in regenerative medicine^30^. Among these, collagen-based scaffolds are the most used in the field of bone regeneration^31^, but a comprehensive *in vivo* study of the bone formation into the implanted biomaterial requires further efforts.

Here, we have characterized *in vivo*, the osteogenic and angiogenic capacity of a novel “bioinspired” collagen/hydroxyapatite biomaterial^8, 10, 12^ after subcutaneous implantation in the mice. The scaffolds were implanted either cell-free or after charging with hADSCs. Then, the time-course of angiogenic and osteogenic processes were analyzed both *in vivo* by Fluorescent Molecular Tomography (FMT), and *ex vivo* by histological analysis.

## Methods

### Scaffold properties

**Figure 1:**
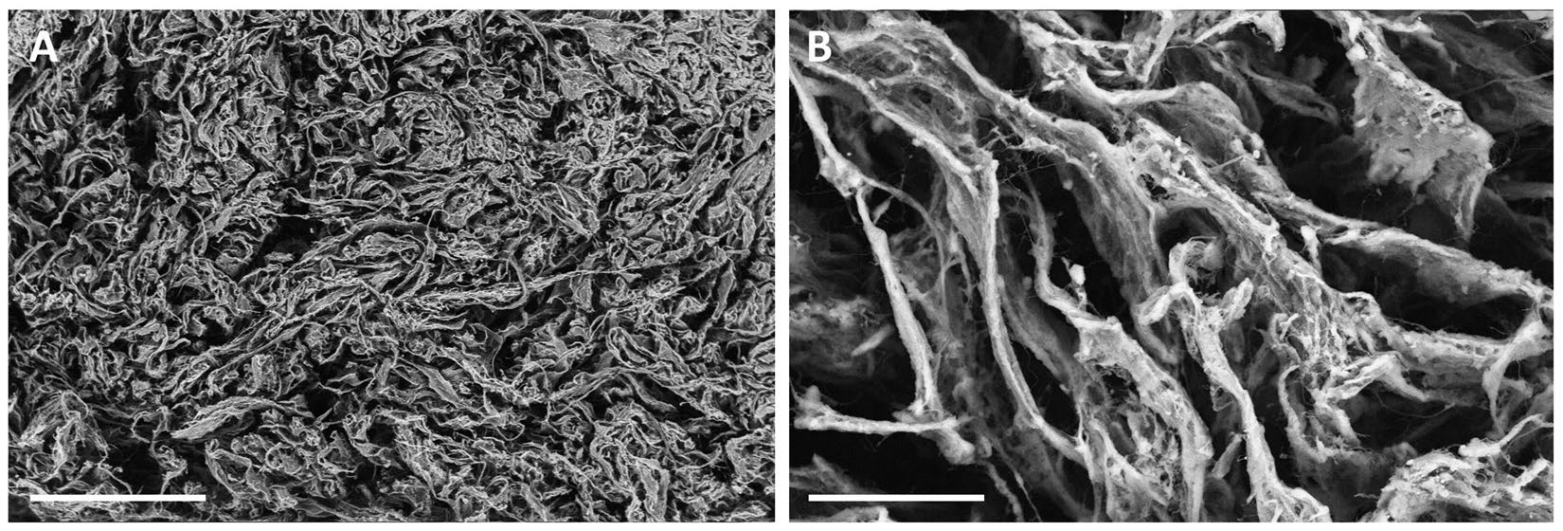
SEM images of the scaffold internal structure at two different magnifications. At higher magnification, an open and interconnected porosity is visible within the bone-like scaffold and Mg-HA particles are visible within and on the surface of the collagen fibers (**B**). Magnification: 30× in A; 300× in B. Scale bars: 1000 µm in (**A**) 100 µm in (**B**).

The biomimetic scaffold used in this study (Fin-Ceramica Faenza SpA, Faenza, Italy) has a cylindric shape, with a 8 mm diameter and 5 mm high, consisting of a mineralized blend of type I collagen (30%) and magnesium-enriched hydroxyapatite (Mg-HA, 70%), reproducing a bone-like tissue^10, 12^ as shown in Fig. 1. The process of fabrication, as well as the chemical and physical characterization and biocompatibility have been described previously^6, 25, 32^.

### Isolation and culture of hADSC

hADSCs were isolated from surgical adipose tissue biopsies obtained from patients, supplied by the Mediterranean Institute of Oncology (IOM) (Viagrande, Italy), under an approved Institutional Review Board protocol (project ID code: 829_1 of 8 February 2013, IOM Institutional Review Board). All patients have provided informed consent concerning the experimental use of their surgical samples. All methods were performed in accordance with the relevant guidelines and regulations. Isolation from adipose tissue biopsies, propagation and characterization were performed as previously described^24, 25, 32, 33^.

Cells were grown in adipose-derived stem cell basal medium with supplements (Lonza Group Ltd., Basel, Switzerland), plated into a 75 cm^2^ flask and incubated at 37 °C and 5% CO_2_. The medium was replaced twice a week until reaching 80% confluence. Then, cells were detached, re-suspended in medium at a density of 5.0 × 10^6^ cells/ml. 200 μl of this cell suspension were charged on each scaffold, placed onto a 12-well plate. The constructs were then placed into the incubator. Two hours later, the scaffolds were submerged in medium and left into the incubator for 24 h more, until subcutaneous implantation in mice.

### Immunofluorescence and flow cytometry analysis of hADSCs

hADSCs characterization was performed by immunofluorescence and flow cytometry. Immunofluorescence analysis was performed on cells seeded in 8-well BD Falcon culture slides at a density of 5 × 10^3^ cells/cm^2^ in adipose-derived stem cell basal medium. The primary incubation was performed, overnight at 4 °C, with the following anti-human antibodies: mouse anti-CD31 (Dilution: 1:100, Santa Cruz Biotechnology, Dallas, TX, USA); rabbit anti-CD34 (1:100; Epitomics, Burlingame, CA, USA); rabbit anti-CD45 (1:100; Epitomics); mouse anti-CD73 (1:25; Novus Biologicals, Littleton, CO, USA); mouse anti-CD105 (1:50; Novus Biologicals) or mouse anti-CD90 (1:50; Santa Cruz Biotechnology). After washing, cells were incubated with the specific secondary Alexa Fluor 568 or 488 antibodies (Life Technologies Italia, Monza, Italy) at the dilution of 1:2000 for 1 h at room temperature. Nuclei were counter stained with DAPI (4′,6-diamidino-2-phenylindole, 1:5,000). Then, slides were mounted in fluorescent mounting medium Permafluor (Thermo Scientific, Waltham, MA, USA) and digital images were acquired using a Leica DMI 4000B fluorescence microscope (Leica, Wetzlar, Germany). Control of immunostaining specificity was performed by omitting the primary antibody.

Flow cytometry was performed on hADSCs at P1. Cells were detached with 0.05% trypsin/EDTA. The cell suspension was subsequently centrifuged and washed in PBS. A total of 1 × 10^4^ cells were incubated with the following antibodies: anti-CD31 PE (Clone 1F11); anti-CD34 PE (Clone 581); anti-CD45 FITC (Clone J.33); anti-CD73 PE (Clone 581); anti-CD105 PE (Clone 1G2); anti-CD90 FITC (Clone F15.42.1.5) and corresponding isotypic controls according to manufacturer indications. All antibodies were purchased from Beckman Coulter (Milano, Italy). All tubes were incubated in the dark for 20 min at room temperature. Cells were then washed with PBS and finally analyzed by flow cytometry, using an FC-500 five-color flow cytometer (Beckman Coulter Inc., Pasadena, CA, USA). For each tube, 1000 events were acquired. CXP Analysis software (Beckman Coulter, Inc.) was used for data analysis.

### Animals and experimental design

Immunodeficient adult female mice (n = 34) (Strain NOD.CB17-Prkdscid/NCrHsd, 4 months aged, weight: 25–30 g; Harlan Laboratories, San Pietro al Natisone, Italy) were used. All experiments involving animals were conducted in accordance with relevant guidelines and regulations. In particular, animal care and handling were carried out in accordance with the EU Directive 2010/63/EU, as well as with the Italian law (D.Lgs. 26/2014). All procedures involving animals were approved by the Italian Ministry of Health. Animals were housed in groups of three in individually ventilated cages (15 changes of filtered air per hour), in dust-free wooden bedding, with *ad libitum* access to water and food (Teklad rodent diet, Harlan Laboratories, San Pietro al Natisone, Italy), with standard conditions of temperature (22 ± 2 °C) and relative humidity (50 ± 5%) and a light/dark cycle of 12/12 h.

Surgical procedures were performed under aseptic conditions, with the animals under gas anaesthesia (isoflurane). Collagen/Mg-HA scaffold with (n = 15) or without hADSCs (n = 15) were implanted into a subcutaneous pocket in the dorsum of animals. Each animal received a single scaffold. All efforts were made to minimize the number of animals used and their suffering. The implanted animals were divided into six groups: 2 wk (Cells, n = 5; No cells, n = 5), 4 wk (Cells, n = 5; No cells, n = 5), 8 wk (Cells, n = 5; No cells, n = 5), and they were sacrificed by CO_2_ inhalation at 2, 4 or 8 weeks after scaffold implantation, respectively, and the scaffolds were collected for analysis. Four additional animals without scaffold implantation were used as negative controls for *in vivo* imaging.

### *In vivo* imaging by FMT

In order to functionally evaluate the occurrence of osteogenesis and angiogenesis within the scaffold in a time-course manner, some animals were analysed by *in vivo* FMT (FMT 2500, Perkin Elmer, Monza, Italy). In particular, all implanted animals, as well as four unoperated mice, received an injection of 100 μl of OsteoSense 750EX (Perkin Elmer), plus 100 μl of AngioSense 680EX (Perkin Elmer) into the tail vein one day before sacrifice. These fluorescent probes specifically bind to newly formed hydroxyapatite and endothelial cells, respectively. Twenty-four hours after the probe injection, FMT images were acquired. During the acquisition, animals were kept under isoflurane anaesthesia and placed into the glass FMT imaging cassette. Acquisition and analysis of FMT images were carried out by using the TrueQuant software (Perkin Elmer). For analysis, the region of interest (ROI) was selected and the extent of osteogenesis or angiogenesis was quantified by measuring the amount of fluorescence probe (in pmol) into the ROI after choosing a concentration threshold. This threshold has been determined by keeping the volume of ROI constant (50 mm^3^). Animals were sacrificed immediately after imaging.

### Histology

Immediately after animal sacrifice, scaffolds were collected and fixed for 2 h in 4% paraformaldehyde at + 4 °C, dehydrated, embedded in paraffin and cut into 3 μm-thick sections. Sections were mounted on slides and stored at room temperature pending staining. After paraffin removal and rehydration, sections were treated for immunohistochemistry (IHC) or stained with Alizarin Red S or hematoxylin and eosin (H&E).

For IHC staining, sections were incubated for 1 h at 25 °C with 5% normal donkey serum and 0.3% Triton  X100 in PBS, and then overnight at 25 °C with one of the following rabbit polyclonal antibodies: anti-alkaline phosphatase (ALPL, LSBio, Seattle, USA; dilution: 1:50); anti-osteocalcin (LSBio; dilution: 1:50); anti-osteonectin (LSBio; dilution: 1:50); anti-osterix (Biorbyt, Cambridge, UK; dilution: 1:250) or anti-CD-31 (Novus Biological, Cambridge, UK; dilution: 1:250). The following day, sections were incubated for 1 h, at room temperature with Alexa Fluor anti-rabbit 568 secondary antibodies (Life Technologies Italia, Monza, Italy; dilution: 1:2000). Then slides were counterstained with DAPI, 1:10000 in PBS for 5 min and mounted with Permafluor. Control of immunostaining specificity was performed by omitting the primary antibody.

Alternate sections were also labeled with H&E and Alizarin Red S, to evaluate the formation of mineralized matrix. For the staining, an Alizarin Red solution was prepared according to manufacturer protocol and sections were incubated for 5 minutes, washed several times and mounted. The stained sections were examined by means of a Leica DMI 4000B microscope (Leica Microsystems Srl, Milano, Italy). At least three serial sections/animal were analyzed. For quantitative evaluation of Alizarin red staining, the optical density (OD) of staining was measured in grayscale images by using Image J (NIH, USA).

### Statistical analysis

Data were analyzed either as raw data or as mean ± SEM, as appropriate. Differences between experimental groups were evaluated by using one-way or two-way ANOVA, where appropriate, followed by Tukey’s or Bonferroni’s *post hoc* test, where appropriate. For all experiments, a P-value of < 0.05 was considered to be significant. All analyses were performed by means of Systat 11 (Systat Software, USA).

### Data availability statement

The datasets generated during and/or analysed during the current study are available from the corresponding author on reasonable request.

## Results

### Phenotypic characterization of hADSCs

**Figure 2:**
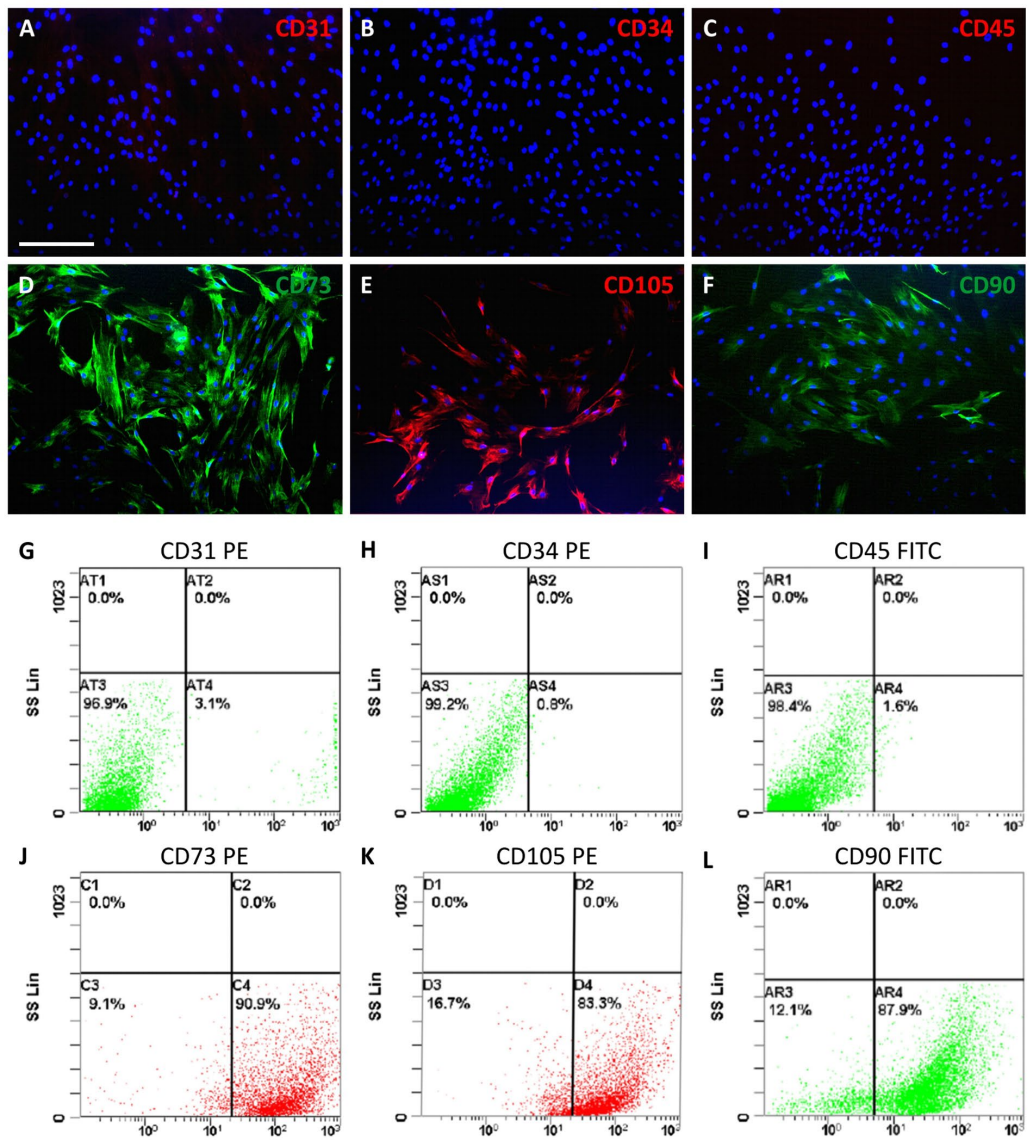
Phenotypic characterization of hADSCs by immunofluorescence and flow cytometry. Different samples were analyzed and, as expected, they didn’t exhibit any expression of CD31 (**A**,**G**), CD34 (**B**,**H**) or CD45 (**C**,**I**), whereas a strong expression of CD73 (**D**,**J**), CD105 (**E**,**K**) and CD90 (**F**,**L**) was found consistently. Scale bar in A for (**A**–**F**) 100 µm.

Immunofluorescence (Fig. 2A–F) and flow cytometry (Fig. 2G–L) have been performed on hADSCs isolated from adipose tissue samples by adhesion to plastic support. Three different hADSC lines were used to study the expression of several typically positive (CD73, CD105, CD90) and negative (CD31, CD34, CD45) surface markers.

The results of both analyses have confirmed that hADSCs were negative for CD31, CD34 and CD45, whereas they exhibited a strong positivity for CD73, CD105 and CD90 (Fig. 2).

### *In vivo* FMT imaging of osteogenesis and angiogenesis within the scaffold

**Figure 3:**
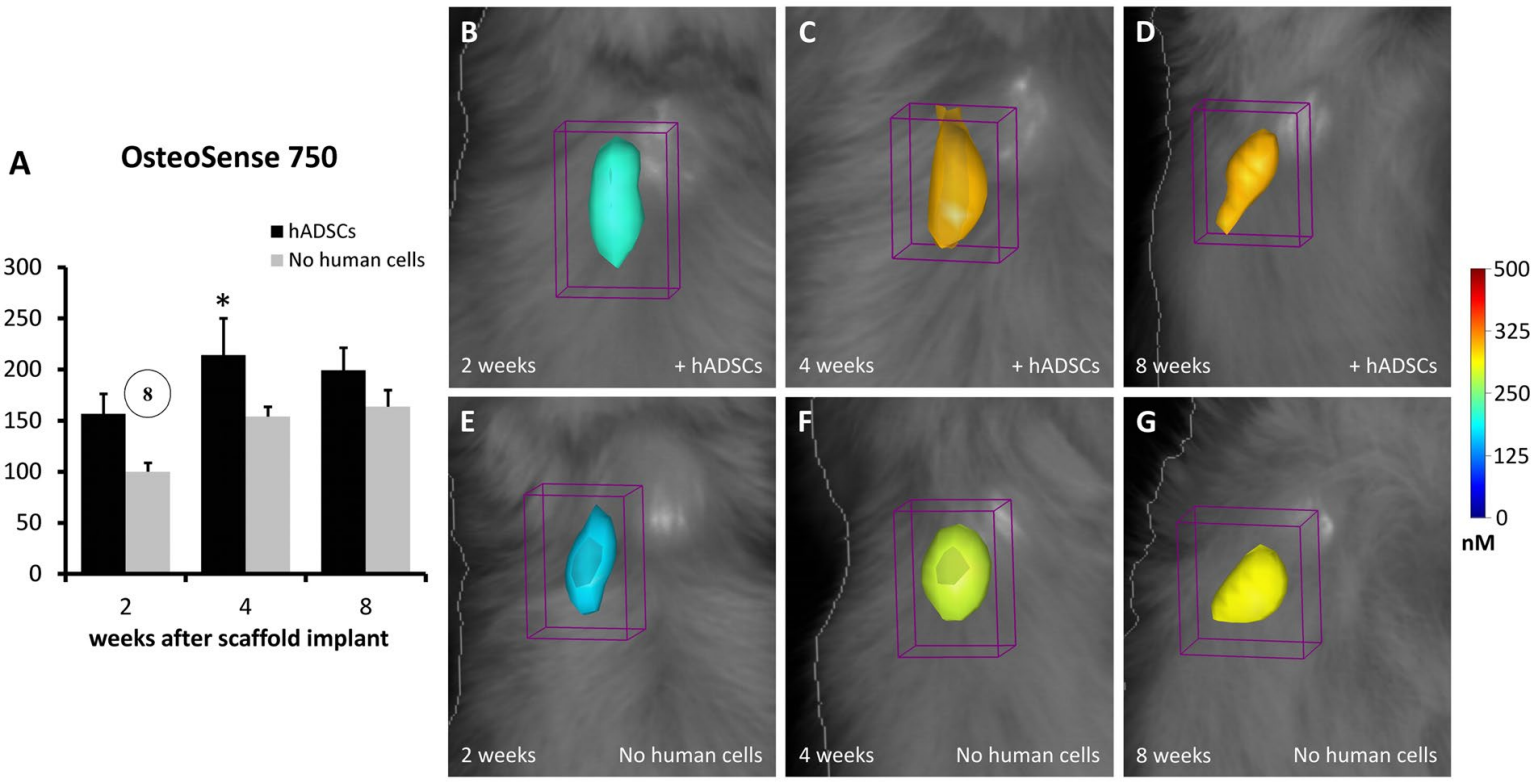
FMT images and *in vivo* quantification of osteogenesis within the implanted scaffolds. The color scale indicates the mean fluorescent probe concentrations within the ROI, and the graph (**A**) shows the mean values of probe content (in pmol) calculated within groups and reported as percent of the mean value of the 2 wk group with cell-free scaffold. It is evident that a higher concentration of probe (OsteoSense 750) is present when the scaffolds contain hADSCs, at all time-points (**B**–**D**) when compared with cell-free scaffolds (**E**–**G**). In the graph A, the number into the circle indicates significant difference (P < 0.05) from the corresponding time-point, in weeks (same treatment group). The asterisk indicates significant difference (P < 0.05) from the other treatment group (same time-point).

The analysis of FMT data has demonstrated the occurrence of osteogenesis within the implanted scaffolds (Fig. 3). In particular, after intravenous injection of OsteoSense 750, the formation of hydroxyapatite has been observed in scaffolds either containing hADSCs or not and increased with time (Two-way ANOVA, effect of time-point: P < 0.05; Fig. 3). Interestingly, the presence of hADSCs significantly quickens the formation of hydroxyapatite (Two-way ANOVA, effect of cell type: P < 0.01; Fig. 3A; compare Fig. 3B–G), especially at 2 and 4 weeks after implantation (Tukey’s post-hoc test: P < 0.05; Fig. 3A).

**Figure 4:**
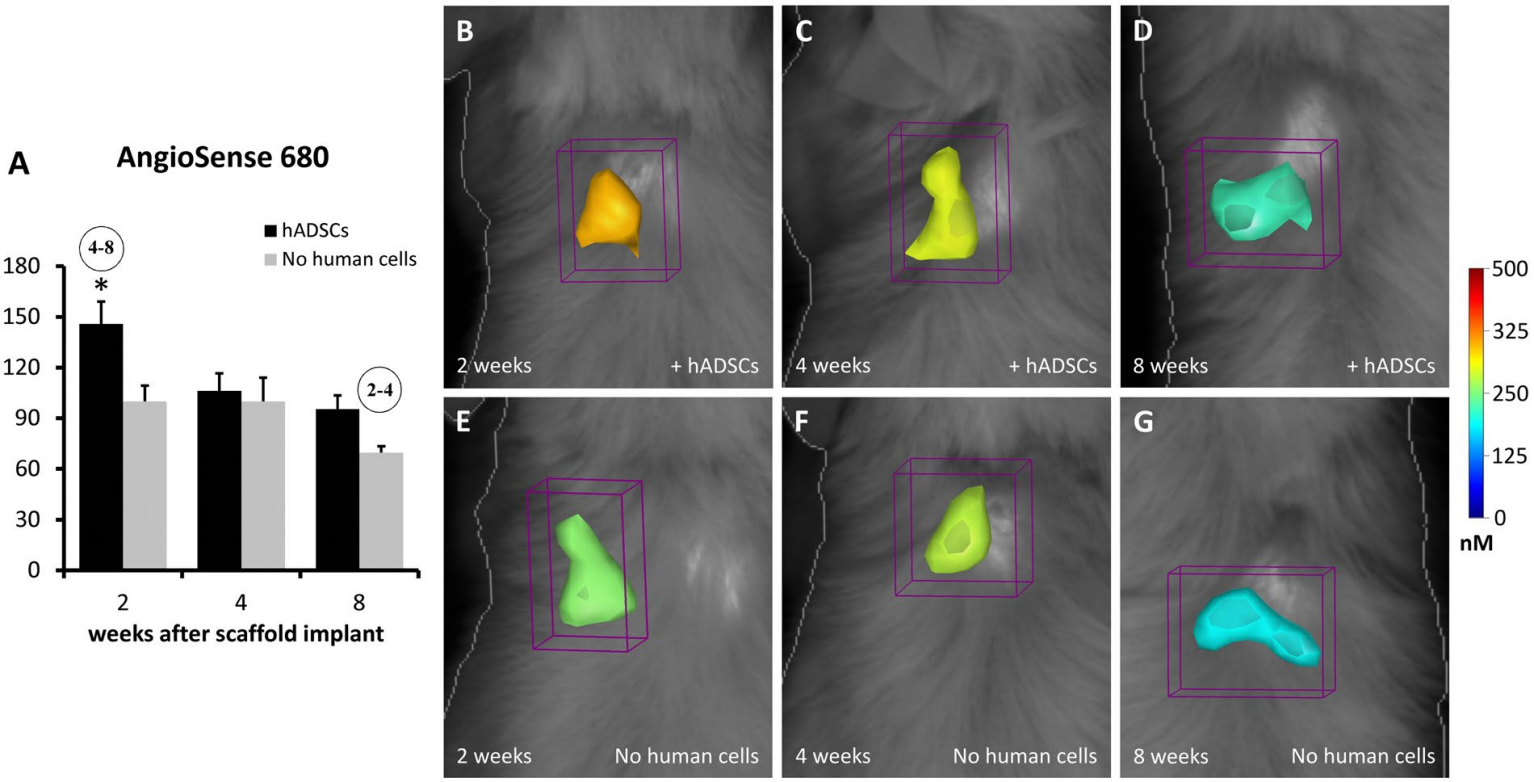
FMT images and *in vivo* quantification of angiogenesis within the implanted scaffolds. The color scale indicates the mean fluorescent probe concentrations within the ROI, and the graph (**A**) shows the mean values of probe content (in pmol) calculated within groups and reported as percent of the mean value of the 2 wk group with cell-free scaffold. A higher concentration of AngioSense 680 is present when the scaffolds contain hADSCs, especially at 2 and 8 weeks (**B**,**D**) when compared with cell-free scaffolds (**E**–**G**). In the graph A, the numbers into the circles indicate significant differences (P < 0.05) from the corresponding time-points, in weeks (same treatment groups), while the asterisk indicates significant difference (P < 0.05) from the other treatment group (same time-point).

Similarly, the Fig. 4 shows the FMT analysis of angiogenesis within the implanted scaffold, *in vivo*. After the intravenous injection of AngioSense 680, the new formation of vascular elements has been observed in both treatment groups, with a decreasing trend along the time-points (Two-way ANOVA, effect of time-point: P < 0.01; Fig. 4). The addition of human cells into the scaffold before implantation have drastically increased the formation of blood vessels, especially at the earlier time-point (Two-way ANOVA, effect of cell type: P < 0.01; Fig. 4A; compare Fig. 4B,E; Tukey’s post-hoc test: P < 0.05; Fig. 4A).

### Histological evaluation of bone augmentation within the scaffold

**Figure 5:**
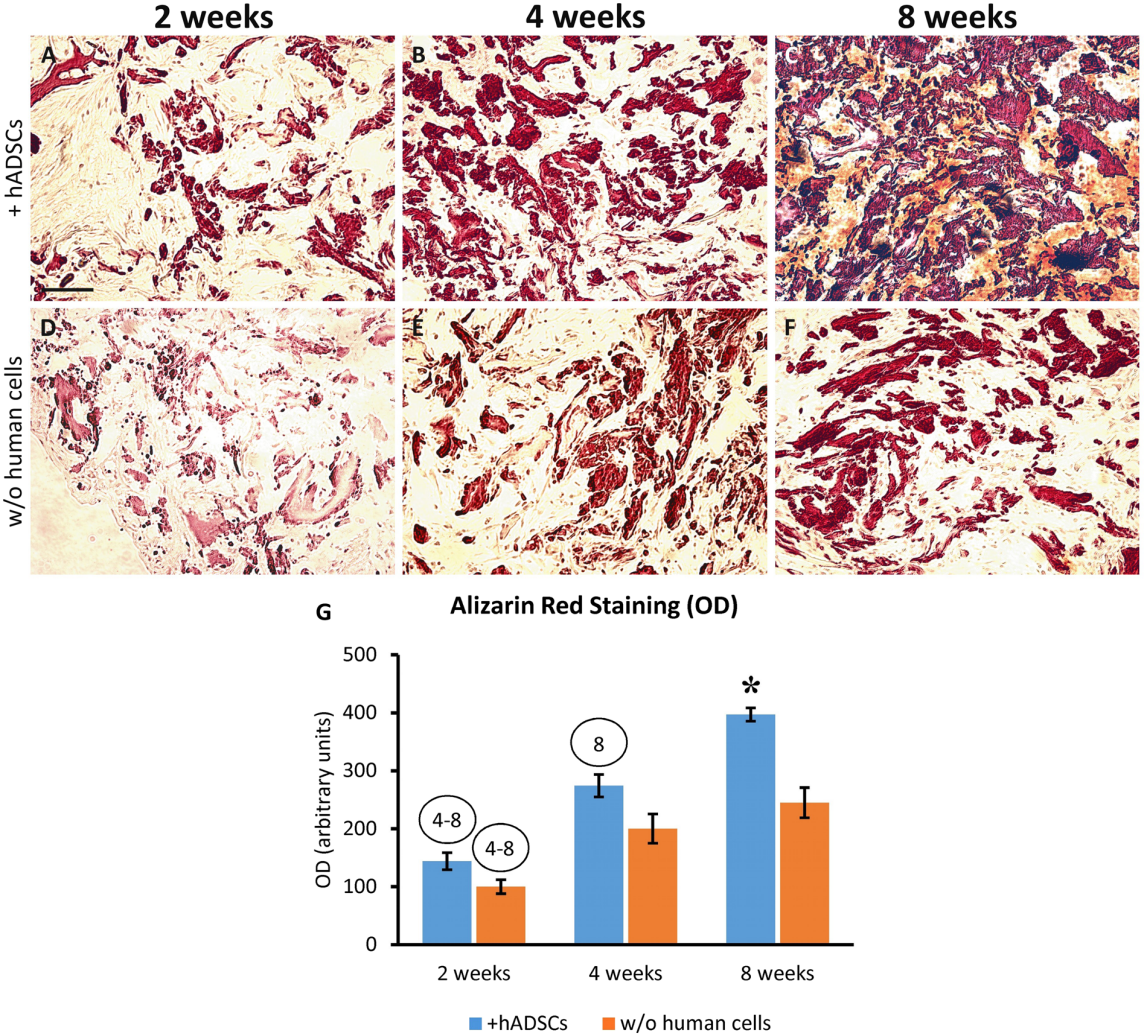
Alizarin Red S staining showing the time-course of mineralization within the scaffolds implanted either charged with hADSCs (**A**–**C**) or cell-free (**D**–**F**) and explanted at 2, 4 or 8 weeks after surgery. It is evident that the mineralization increased overtime in both treatment groups, but the addition of hADSCs determined a strong increase of this process. The graph (**G**) shows the quantitative evaluation of Alizarin Red staining by measuring the optical density (OD). The numbers into the circles indicate significant differences (P < 0.05) from the corresponding time-points, in weeks (same treatment groups), while the asterisk indicates significant difference (P < 0.05) from the other treatment group (same time-point). Scale bar: 100 μm.

In order to assess the formation of calcium deposits and mineralized matrix, Alizarin Red S staining was used. The staining has revealed that the mineralization gradually increased from 2 to 8 week, in scaffolds either with or without hADSCs (Fig. 5), although the presence of hADSCs strongly increased the process, thus resulting in a stronger Alizarin Red staining (Fig. 5A–C) as compared to the scaffolds sections without hADSCs (Fig. 5D–F). The quantitative evaluation of Alizarin Red staining was carried out by measuring the OD in grayscale images (Fig. 5G). It is evident that mineralization increased overtime (two-way ANOVA: P < 0.001). However, the presence of hADSCs increased the process (two-way ANOVA: P < 0.05), especially at the longer time-point (Bonferroni’s *post hoc* test: P < 0.05).

**Figure 6:**
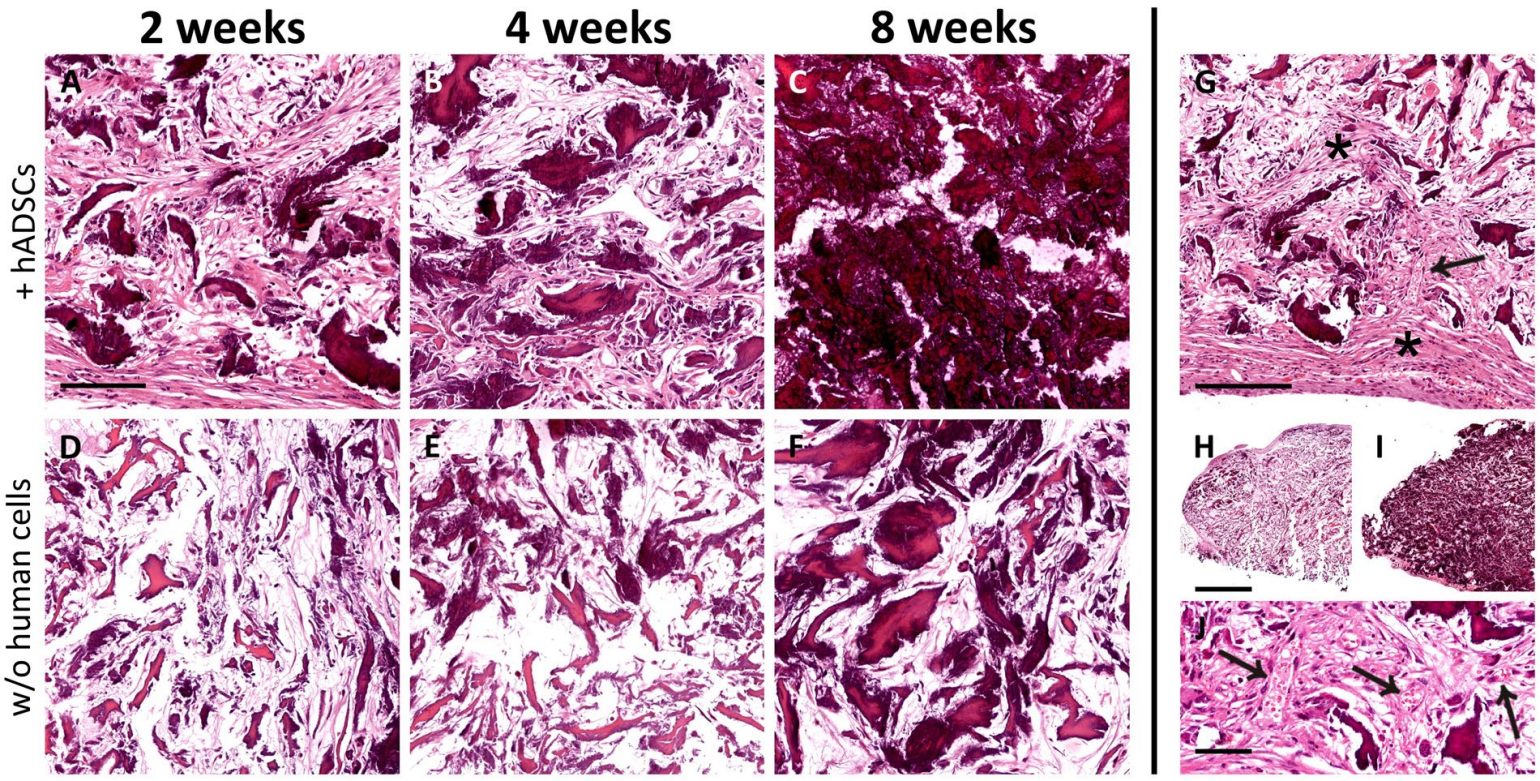
Time-course of tissue modifications observed by Hematoxylin and Eosin staining in the scaffolds explanted at 2 (**A**,**D**), 4 (**B**,**E**) or 8 (**C**,**F**) weeks after subcutaneous implantation, either with (**A**–**C**) or without (**D**–**F**) the addition of hADSCs. It is evident that the material was wrapped, and then invaded, by fibroblast-like cells (asterisks in **G**), and this process was more robust in scaffolds seeded with hADSCs (compare **A**,**B** with **D**,**E**). Along the observed time-points, the material undergone a visible mineralization that, again, appeared dramatically increased in scaffolds seeded with hADSCs prior to implantation (compare **A**–**C** with **D**–**F**). In particular, the scaffold appeared almost completely mineralized at 8 weeks (**C**). The mineralization is evident also comparing the scaffolds sections at low magnification in H (2 weeks, without hADSCs) and I (8 weeks, with hADSCs). The formation of vascular elements is evident in G (arrow) and at higher magnification in J (arrows). Scale bar in A for A-F: 100 μm; in G: 200 μm; in H for H, I: 1 mm; in J: 50 μm.

H&E staining showed that the scaffolds undergone a striking transformation after implantation (Fig. 6). Since the second week after implantation, an evident *de novo* mineralization occurred within the biomaterial either in presence or absence of hADSCs, and this process constantly increased overtime, as showed by the formation of dark areas within the tissue. However, the bone augmentation process appeared much stronger when the biomaterial was previously charged with hADSCs (compare Fig. 6A–C with Fig. 6D–F) and, at 8 weeks post-implantation, the scaffold appeared completely mineralized (Fig. 6C; see also Fig. 6H,I for a comparison between the 2 week and 8 week time-points). As shown in Fig. 6G, the scaffolds were wrapped and invaded by fibroblast-like cells (asterisks), and this process was particularly evident in the scaffolds plus hADSCs (Fig. 6A,B), as early as the second week after implantation (Fig. 6A).

Moreover, H&E staining confirmed the data obtained by FMT analysis regarding the vascularization of the scaffolds (Fig. 6G,J, arrows). In particular, vascular elements were more abundant at 4 and 8 weeks post-implantation.

**Figure 7:**
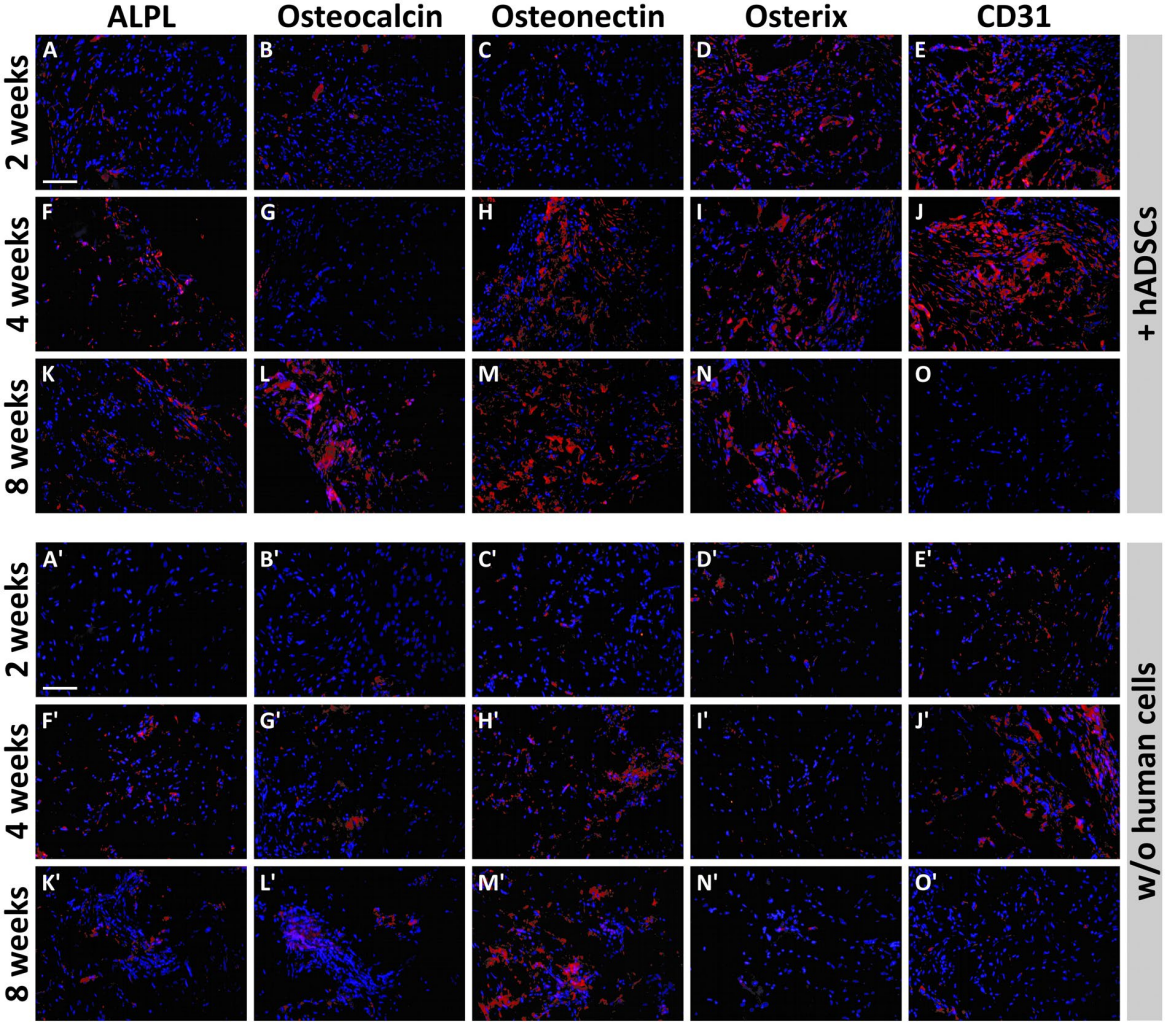
Panel of fluorescence microscope images showing the time-course of expression of osteogenic and angiogenic markers in scaffolds with or without hADSCs (red fluorescence). It is evident that the expression of some osteogenic markers in scaffolds plus hADSCs, especially ALPL (**A**,**F**,**K**), Osteocalcin (**B**,**G**,**L**) and Osteonectin (**C**,**H**,**M**) is increasing with time, whereas the expression of Osterix was constant along time-points (**D**,**I**,**N**) and CD31 appeared higher at 2-4 weeks post-implantation (**E**,**J**) and then decreased at the longer time-point (**O**). A similar pattern of time-course expression appears in scaffolds without human cells. In particular, the expression of ALPL (**A’**,**F’**,**K’**) and Osteonectin (**C’**,**H’**,**M’**) is increasing with time, whereas the expression of CD31 appeared higher at 2-4 weeks post-implantation (**E’**,**J’**) and then decreased at the longer time-point (**O’**). It is also evident that the expression of all analysed markers seems to be lower than that observed in scaffolds containing hADSCs. Nuclei have been stained with DAPI (blue). Scale bars: 100 μm.

**Figure 8:**
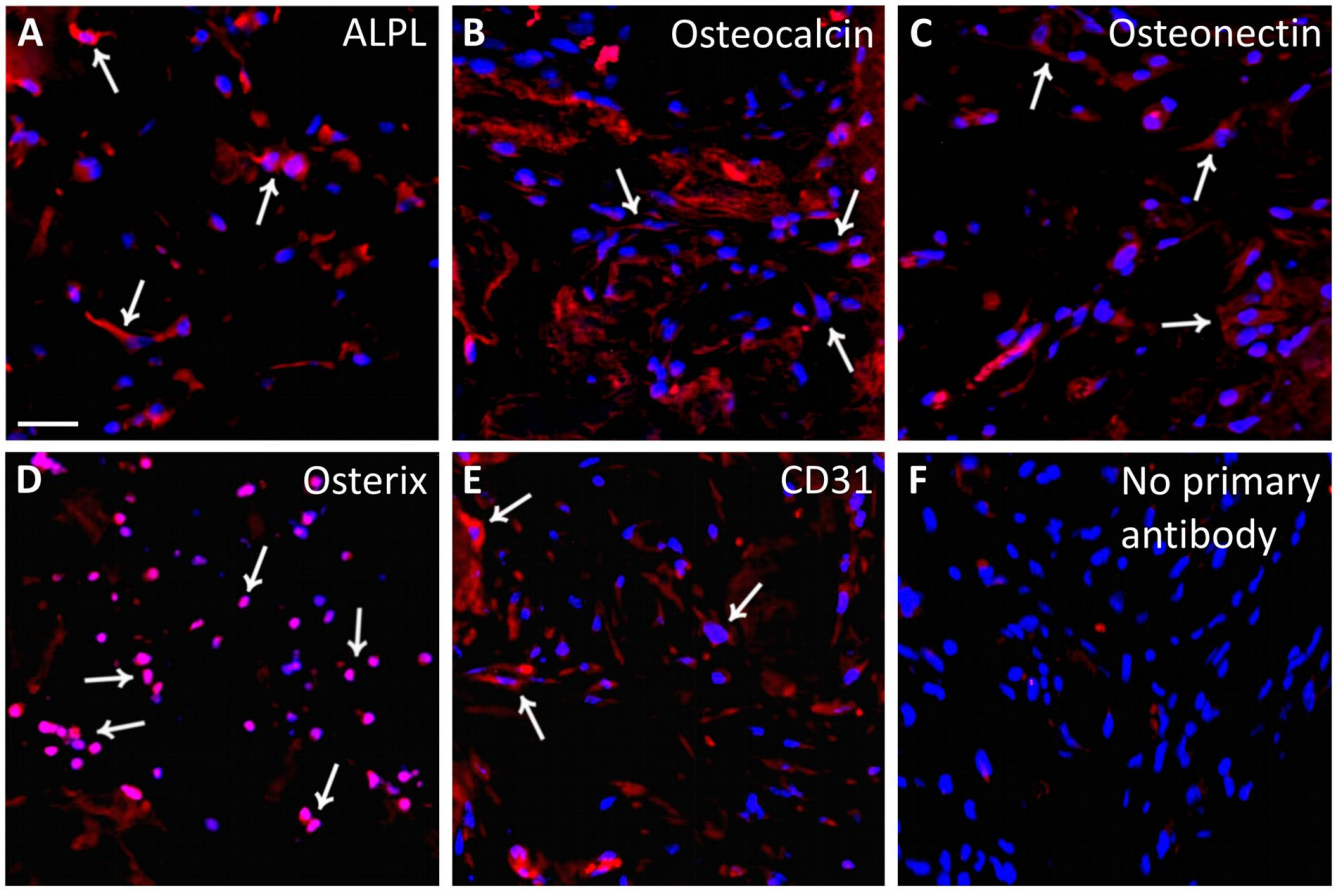
Panel of fluorescence microscope images showing typical examples of ALPL (**A**), Osteocalcin (**B**), Osteonectin (**C**), Osterix (**D**) and CD31 (**E**) expression (red fluorescence). Arrows indicates some positive cells. A negative control obtained by omitting the primary antibody shows the absence of any fluorescent signal. Nuclei have been stained with DAPI (blue). Scale bar: 40 μm.

Bone augmentation and angiogenesis within the implanted scaffolds, either alone or seeded with hADSCs, were also evaluated by immunofluorescence by analyzing several markers of osteogenic differentiation, including ALPL, Osteocalcin, Osteonectin and Osterix, as well as CD31 that is a specific vascular marker. ALPL, which is one of the earliest markers of bone formation, displays a stronger signal in the scaffolds containing hADSCs at every time-point after implantation (Fig. 7A,F,K), as compared to the scaffolds without human cells (Fig. 7A’, F’, K’). Osteocalcin, which is a typical late osteoblast marker, exhibits no or a weak signal during the first four weeks after implantation in scaffolds either with or without hADSCs (Fig. 7B,G and Fig. 7B’,G’), that increased at week 8, especially in scaffolds seeded with hADSCs (Fig. 7L). Osteonectin, which is an acidic glycoprotein of the extracellular matrix and plays a crucial role in bone mineralization, was weakly expressed during the first two weeks (Fig. 7C and Fig. 7C’), but increased at 4 and 8 weeks, especially in presence of hADSCs (compare Fig. 7H,M with Fig. 7H’,M’). IHC analysis of Osterix that is an early marker of osteoblast differentiation, exhibits no or a very weak signal at every time-point in the scaffolds without hADSCs (Fig. 7D’,I’,N’), whereas a signal appeared in presence of hADSCs, remaining virtually constant at all time-points (Fig. 7D,I,N). The expression of the CD31 endothelial marker was in line with the results of FMT imaging and H&E staining, showing a weak CD31 signal at the second week (Fig. 7E and Fig. 7E’) with an expression peak at week 4 that was stronger in presence of hADSCs (compare Fig. 7J and Fig. 7J’). Conversely, CD31 expression appeared markedly reduced at week 8 (Fig. 7O and Fig. 7O’). Higher magnification images of immunostaining (Fig. 8) better show the pattern of protein expression in cells as membrane or cytoplasmic (Fig. 8A,B,C,E), or nuclear signal (Fig. 8D). Negative controls have been performed by omitting the primary antibodies and they did not show any significant fluorescent signal (Fig. 8F).

## Discussion

Repairing large bone injuries is still a big challenge in orthopedics and the possible use of biomimetic materials capable of improving the restoration of bone structure and function represents a promising clinical approach. A novel bioinspired collagen/Mg-HA scaffold has been previously studied in terms of physicochemical properties^6, 10, 12, 25, 32^, as well as in terms of its ability to incorporate mesenchymal stem cells and commit them to the differentiation towards the osteogenic or chondrogenic lineage *in vitro* ^10, 12, 25, 32^. Moreover, *in vivo* experiments carried out in our laboratory have proven that this biomaterial is able to induce bone augmentation and angiogenesis after ectopic implantation in the mouse, by the recruitment of host cells that are able to populate the scaffold^6^. Previous *in vitro* and *in vivo* evaluations have also demonstrated that the material is safe, and no significant signs of inflammation have been found even when the material was subcutaneously implanted in immunocompetent mice^6^.

In the present study, we made a comparative study of this biomaterial to evaluate its capacity of inducing ectopic bone formation *in vivo*, after subcutaneous transplantation in the mouse dorsum either as a cell-free scaffold or after seeding it with hADSCs. All animals remained healthy for the entire period of the study, showing no evident sign of toxicity or other adverse effects. Scaffold mineralization as well as the formation of vascular structures have been evaluated *in vivo* in a time-course manner, by using the FMT technology. These *in vivo* studies, together with the *ex vivo* histological evaluation of the scaffolds, have firstly demonstrated that the biomaterial is able to recruit host cells and commit them towards osteo- and angio-genesis, thus inducing a spontaneous bone augmentation and confirming previous findings^6, 34^. The formation of well-organized blood vessels within the biomaterial is an important finding because it indicates a good graft-host interaction and represents an important condition for the long-term cell survival. In fact, for a successful bone repair, osteogenesis should be accompanied by the vascularization of the biomaterial. This process has been shown *in vivo* by FMT analysis and confirmed histologically, as well as by the expression of CD31. However, the number of blood vessels seems to be reduced at eight weeks, probably because of the increasing formation of new mineralized tissue. The elucidation of the mechanisms underlying the formation of vascular elements requires further investigation but, however, similar results were found by other authors, showing that either bone marrow- or adipose-derived MSCs could have angiogenic capacity when seeded into different biomaterials^14, 15^.

Although the spontaneous bone augmentation is possible by means of the endogenous stem cells, this process could be less efficient in aged patients or in other particular group of patients, including for instance those subjected to chemotherapy. For this reason, we sought to determine if the bone formation could be improved by the addition of human cells. In particular, we have used hADSCs for their *in vitro* properties, ease of withdrawal and expansion for autologous transplantation. *In vitro* experiments in our laboratory have proven that these cells have higher capacity of proliferation and tri-lineage differentiation when compared to those derived from bone marrow stroma^24^. Other *in vitro* studies in combination with collagen/Mg-HA scaffolds have proven their ability to populate the biomaterial and undergone osteogenic differentiation and scaffold mineralization, even without the addition of osteo-inductive factors to the cell medium^25, 32^. Here, our data have shown that the addition of hADSCs to the scaffold prior to the subcutaneous implantation significantly improved both mineralization and vascularization. *In vivo* analysis by FMT, in fact, has proven that the presence of hADSCs could increase the formation of hydroxyapatite and vascular elements, and these results are confirmed by the histological evidence of improved calcification of the scaffold material, seen after Alizarin Red or H&E staining. In particular, FMT analysis showed that the formation of hydroxyapatite was dramatically increased especially during the first four weeks after implantation, and this process caused an evident accumulation of calcified matrix, which was particularly evident at the later time points. Our previous results, as well as similar published results^34^, have demonstrated that a collagen/Mg-HA scaffold can be spontaneously mineralized *in vivo*. These results, along with those reported here, demonstrate that the ability of inducing migration and differentiation of resident or grafted cells is an intrinsic property of the biomaterial, probably linked to the Mg-HA content^6^, as also reported in other published papers^34–36^. Collagen, which is the main protein component of bone, is able to induce MSCs differentiation into osteoblasts^35^. Moreover, it has been shown that cell proliferation and differentiation, as well as bone matrix formation are improved within biomaterials containing hydroxyapatite when compared to scaffolds without hydroxyapatite^36^. As occurring in many transplantation models, the local tissue microenvironment is an important factor affecting cell behavior, along with the biomaterial composition and intrinsic properties of cells^6, 24, 25, 32, 37^. Therefore, further studies should address this question by implanting the scaffolds into other organs, including injured bone sites. The orthotopic grafting of this biomaterial, either with or without the addition of human cells is expected to produce a different organization of the newly formed mineralized tissue.

*Ex vivo* molecular characterization of explanted samples confirms that the cells populating the scaffolds were correctly committed towards bone formation through the well-known molecular pathways^38–42^ that involve the expression of markers such as ALPL, osteocalcin, osteonectin and osterix. Interestingly, the expression of these markers appears markedly increased in the scaffolds seeded with hADSCs prior to the implantation.

In conclusion, our data demonstrate that a collagen/Mg-HA scaffold subcutaneously implanted in mice is able to recruit host cells that invade the material, undergo osteogenic differentiation and induce bone augmentation and formation of vascular elements. Notably, the addition of hADSCs to the scaffolds prior to implantation strongly improves these processes. Given the demonstrated safety of this biomaterial, the use of scaffolds alone could represent a safe and promising approach for bone healing, without additional use of growth factors. The use of autologous hADSCs in combination with the scaffolds represents a feasible and promising clinical choice, and some recent studies confirm our findings, although in combination with biomaterials different from that used here^26–29^. In particular, the use of hADSCs along with scaffolds would be promising especially for elderly individuals or other particular groups of patients with reduced regenerative capacity. However, further studies should better characterize the scaffold properties in different tissue environments, including lesioned bone.
